# Incomplete and Inaccurate Vocal Imitation after Knockdown of *FoxP2* in Songbird Basal Ganglia Nucleus Area X

**DOI:** 10.1371/journal.pbio.0050321

**Published:** 2007-12-04

**Authors:** Sebastian Haesler, Christelle Rochefort, Benjamin Georgi, Pawel Licznerski, Pavel Osten, Constance Scharff

**Affiliations:** 1 Max-Planck-Institute for Molecular Genetics, Berlin, Germany; 2 Institut für Verhaltensbiologie, Freie Universität Berlin, Berlin, Germany; 3 Max-Planck-Institute for Medical Research, Heidelberg, Germany; 4 Feinberg School of Medicine, Northwestern University, Chicago, Illinois, United States of America; Duke University Medical Center, United States of America

## Abstract

The gene encoding the forkhead box transcription factor, *FOXP2*, is essential for developing the full articulatory power of human language. Mutations of *FOXP2* cause developmental verbal dyspraxia (DVD), a speech and language disorder that compromises the fluent production of words and the correct use and comprehension of grammar. *FOXP2* patients have structural and functional abnormalities in the striatum of the basal ganglia, which also express high levels of *FOXP2*. Since human speech and learned vocalizations in songbirds bear behavioral and neural parallels, songbirds provide a genuine model for investigating the basic principles of speech and its pathologies. In zebra finch Area X, a basal ganglia structure necessary for song learning, *FoxP2* expression increases during the time when song learning occurs. Here, we used lentivirus-mediated RNA interference (RNAi) to reduce FoxP2 levels in Area X during song development. Knockdown of *FoxP2* resulted in an incomplete and inaccurate imitation of tutor song. Inaccurate vocal imitation was already evident early during song ontogeny and persisted into adulthood. The acoustic structure and the duration of adult song syllables were abnormally variable, similar to word production in children with DVD. Our findings provide the first example of a functional gene analysis in songbirds and suggest that normal auditory-guided vocal motor learning requires *FoxP2.*

## Author Summary

Do special “human” genes provide the biological substrate for uniquely human traits, such as language? Genetic aberrations of the human *FoxP2* gene impair speech production and comprehension, yet the relative contributions of *FoxP2* to brain development and function are unknown. Songbirds are a useful model to address this because, like human youngsters, they learn to vocalize by imitating the sounds of their elders. Previously, we found that when young zebra finches learn to sing or when adult canaries change their song seasonally, FoxP2 is up-regulated in Area X, a brain region important for song plasticity. Here, we reduced FoxP2 levels in Area X before zebra finches started to learn their song, using virus-mediated RNA interference for the first time in songbird brains. Birds with experimentally lowered levels of FoxP2 imitated their tutor's song imprecisely and sang more variably than controls. FoxP2 thus appears to be critical for proper song development. These results suggest that humans and birds may employ similar molecular substrates for vocal learning, which can now be further analyzed in an experimental animal system.

## Abstract 3

The *FoxP2* gene, which is essential for human speech and language, is also required for proper song development in songbirds, raising the possibility that songbirds and humans share molecular pathways for learned vocalizations.

## Introduction

Genetic aberrations of *FOXP2* cause developmental verbal dyspraxia (DVD), which is characterized by impaired production of sequenced mouth movements and both expressive and receptive language deficits [1–4]. Brain imaging studies in adult *FOXP2* patients implicate the basal ganglia as key affected regions [5–7], and *FOXP2* is prominently expressed in the developing human striatum [8]. These findings raise the question whether the speech and language abnormalities observed in individuals with DVD result from erroneous brain development or impaired function of differentiated neural circuits in the postnatal brain, or a combination of both. Human speech and learned vocalizations in oscine birds bear behavioral and neural parallels [9]. Thus songbirds are a suitable model for studying the neural mechanisms of imitative vocal learning, including speech and its pathologies. The *FoxP2* expression patterns in songbird and human brains are very similar, with strong expression in the basal ganglia, thalamus, and cerebellum [8,10,11]. Moreover, *FoxP2* expression in the basal ganglia song nucleus, Area X, which is important for normal song development [12,13], transiently increases at the time when young zebra finches learn to sing. In adult canaries, *FoxP2* expression in Area X is elevated during the late summer months, coincident with the incorporation of most new syllables to their seasonally changing song [10]. *FoxP2* is down-regulated in Area X when adult zebra finches sing slightly variable, undirected song, but not when they sing more stereotyped female-directed song [14]. Together, these correlative findings raise the question whether *FoxP2* and vocal plasticity are causally related.

Using lentivirus-mediated RNA interference (RNAi) during song development, we now show that zebra finches with reduced FoxP2 expression levels in Area X imitated tutor songs incompletely and inaccurately. This effect was already evident during vocal practice in young birds. Moreover, the acoustic structure and the duration of song syllables in adults were abnormally variable, similar to word production in children with DVD [15]. These findings are consistent with a role of *FoxP2* during auditory-guided vocal motor learning in songbird basal ganglia.

## Results

### Establishing Lentiviral-Mediated RNAi in the Zebra Finch

Vocal learning in zebra finches proceeds through characteristic stages. In the sensory phase that commences around 25 d after hatching (post-hatch day [PHD]), young males memorize the song of an adult male tutor. Concomitantly, they start vocalizing the so called “subsong,” consisting of quietly uttered, poorly articulated, and nonstereotypically sequenced syllables [16]. Following intensive vocal practice and improvement toward matching the tutor song during the period of “plastic song,” they eventually imitate the song of their tutor with remarkable fidelity around PHD90. The structural and temporal characteristics of adult “crystallized” song remain essentially stable throughout adult life. To study the function of *FoxP2* during song learning of zebra finches, we reduced the levels of *FoxP2* expression bilaterally in Area X in vivo, using lentivirus-mediated RNAi. In this approach, short interfering hairpin RNA (shRNA) containing sense and antisense sequences of the target gene connected by a hairpin loop are expressed from a viral vector. The virus stably integrates into the host genome, enabling expression throughout the life of the animal [17].

**Figure 1:**
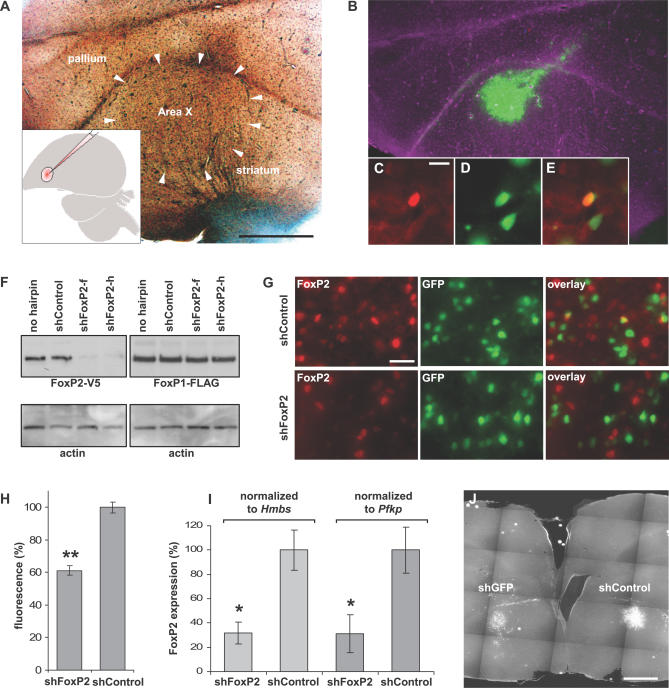
Establishing Lentivirus-Mediated Knockdown of *FoxP2* in Zebra Finch Area X (A) Phase contrast image of a sagittal 50-μm brain section from a male zebra finch. Area X is outlined by white arrows (scale bar indicates 1 mm). The microinjection into Area X is schematized in the inset. (B) Fluorescent microscopy image of (A). Virus-infected cells expressed GFP (green). (C) FoxP2 immunostaining (red; scale bar indicates 10 μm) (D) The neuron shown in (C) also expressed viral GFP from injection with the nontargeting shControl virus. (E) Overlay picture of (C) and (D). (F) Overexpression of zebra finch FoxP2 (left panel) or FoxP1 (right panel), each tagged with the V5 epitope, and one of different hairpin constructs (shFoxP2-f, shFoxP2-h, or shControl) in HEK293 T cells. Western blot analysis using a V5 antibody revealed that shFoxP2-f and shFoxP2-h, but not shControl, efficiently reduced *FoxP2* levels. FoxP1 protein levels were unaffected by overexpression of either shRNA. Immunostaining with an actin antibody shows comparable loading of protein samples. (G) Knockdown of *FoxP2* in vivo. Immunofluorescent staining with an antibody against FoxP2 on 50-μm brain sections from birds injected with shFoxP2-f/-h in one hemisphere and shControl in the contralateral hemisphere 30 d prior to analysis revealed lower fluorescence levels and fewer cells in knockdown (upper panel) compared to control sections (lower panel). FoxP2-positive cells appear red; virally infected cells express GFP, visible in green (scale bar indicates 20 μm). (H) Quantification of in vivo knockdown efficiency. The fluorescence intensity of FoxP2 immunostaining was measured in images from brain sections injected with shFoxP2-f/-h in one hemisphere and shControl in the contralateral hemisphere 30 d prior to analysis. All antibody incubations were performed simultaneously, and pictures were taken with identical camera settings. Bars represent average intensity levels normalized to the shControl-injected hemisphere (± standard error of the mean [SEM]; two-tailed Mann-Whitney *U* test, **p < 0.003; *n* = 4 animals [2 images per hemisphere]). (I) Real-time PCR quantification of *FoxP2* mRNA expression in Area X on PHD50. Animals were injected with shControl in one hemisphere and shFoxP2 virus in the contralateral hemisphere, on PHD23. Bars represent relative gene expression between shControl- and shFoxP2-injected hemispheres, normalized to either *Hmbs* or *Pfkp* as indicated (±STDEV; two-tailed Mann-Whitney *U* test, *p < 0.008; *n* = 5 animals). (J) RNAi-mediated knockdown persisted for at least 3 mo. Frontal 50-μm brain slice of zebra finch injected with the indicated virus 105 d prior to perfusion. The intensity of *GFP* expression, visible as white signal, was lower in the left hemisphere injected with the virus targeting *GFP*, compared to the hemisphere that was injected with shControl (scale bar indicates 1 mm).

We designed two different shRNAs (shFoxP2-f and shFoxP2-h) targeting different sequences in the *FoxP2* gene. Both hairpins strongly reduced the levels of overexpressed FoxP2 protein in vitro (Figure 1F), but did not change the levels of overexpressed protein levels of *FoxP1*, the closest homolog of *FoxP2*. For further control experiments, we generated a shRNA designed not to target any zebra finch gene (shControl). As expected, this nontargeting shRNA did not affect expression of either *FoxP2* or *FoxP1* in vitro (Figure 1F). Since shFoxP2-f and shFoxP2-h targeted *FoxP2* with similar efficiency, both of them were interchangeably used for subsequent in vivo experiments (shFoxP2-f/-h).

On PHD23, at the onset of sensory-motor learning [16] we injected either *FoxP2* knockdown or control viruses (shControl and shGFP; see below) stereotaxically into Area X to achieve spatial control of knockdown (Figure 1A). Starting on PHD30, each young bird, here called pupil, was kept in a sound isolation chamber, together with an adult male zebra finch as tutor. At PHD65, PHD80, and between PHD90 and PHD93, we recorded the pupils' vocalization for subsequent song analysis (for timeline of experiments, see Figure S1). After the last song recording, brains were histologically analyzed for correct targeting of virus to Area X. All lentiviral constructs expressed the green fluorescent protein (*GFP*) reporter gene, allowing the detection of infected brain areas by fluorescence microscopy (Figure 1B). On average, 20.3% ± 9.9% (mean ± standard deviation [STDV]; *n* = 24 hemispheres from 12 animals) of the total volume of Area X was infected. Importantly, there was no difference in the volume of Area X targeted with *FoxP2* knockdown or control viruses (two-tailed Mann-Whitney *U* test, *p* > 0.5; shFoxP2-f/-h, *n* = 6, shControl, *n* = 7). Quantification of Area X volume targeted by virus injection in an equally treated group of birds, but sacrificed at PHD50, confirmed the results obtained for PHD90 (mean volume 20.4% ± STDV 4.0%; two-tailed Mann-Whitney *U* test, *p* > 0.6; shControl *n* = 3 hemispheres from 3 animals; shFoxP2-f/-h, *n* = 3 hemispheres from 3 animals).

To quantify the neuronal extent of lentivirus expression in Area X, we used immunohistochemical staining with the neuronal marker Hu [18] (Figure S2). Of all virus-infected cells, 78.5% ± 3.5% were neurons (mean ± standard error of the mean [SEM]; no significant difference between shFoxP2 and shControl, two-tailed Mann-Whitney *U* test, *p* > 0.7; shControl injections *n* = 3 hemispheres from 3 animals, shFoxP2 injections *n* = 4 hemispheres from 4 animals;). This result is consistent with Wada et al. [19], who used the same viral constructs in the zebra finch brain in vivo. Among the infected cells were FoxP2-positive spiny neurons, which are assumed to be the most common cell type in Area X [20] (Figure 1C–1E).

To quantify *FoxP2* knockdown in vivo, we determined FoxP2 protein levels in Area X on PHD50, the time of peak *FoxP2* expression [10] in birds injected on PHD23 with shFoxP2-f/-h in one hemisphere and shControl into the contralateral hemisphere. The signal of the immunofluorescent staining with a FoxP2 antibody was significantly lower in knockdown Area X than in control Area X (Figure 1G and 1H). We also assessed *FoxP2* mRNA levels after knockdown in Area X. Birds were injected on PHD23 with shFoxP2-f/-h in one hemisphere and shControl in the contralateral hemisphere. On PHD50, we punched out Area X of injected birds and measured *FoxP2* mRNA levels by real-time PCR. *FoxP2* levels were normalized to two independent RNAs coding for the housekeeping genes *Hmbs* and *Pfkp*. *FoxP2* mRNA was reduced on average by approximately 70% in the shFoxP2-infected region of Area X compared to the shControl-infected region of Area X (Figure 1I). Of note, RNAi-mediated knockdown approximates FOXP2 levels in DVD patients, since haploinsufficiency, a 50% reduction of functional FOXP2 protein, is apparently the common feature of all reported human *FOXP2* mutations [4,21].

To demonstrate that RNAi-mediated gene knockdown can persist in vivo throughout the entire song-learning phase, we used a virus expressing shRNA against the viral reporter *GFP* (shGFP) in conjunction with the virus expressing a shRNA lacking a target gene (shControl). We injected young zebra finches on PHD23 with equal amounts of equally infectious shGFP and shControl virus in the left and right hemisphere, respectively. More than 3 mo later, on PHD130, the GFP signal in the shGFP-injected hemisphere was still 70.5% ± 5.8% less intense than in the shControl-injected hemisphere (mean ± SEM; *n* = 2; Figure 1J).

To rule out potential side effects of *FoxP2* knockdown on cellular survival in Area X, we investigated apoptosis in Area X 6 d after surgery with terminal deoxyribonucleotide transferase-mediated dUTP nick end labeling (TUNEL). The TUNEL method detects genomic DNA double-strand breaks characteristic of apoptotic cells. Of 1,149 GFP-positive cells counted in six hemispheres from three animals, only five were TUNEL-positive (Figure S3). ShControl-injected and uninjected animals had similar low levels of apoptotic cells (unpublished data). Thus, *FoxP2* is not a gene essential for short-term survival of postmitotic neurons. Since the TUNEL method does not capture any long-term changes in neuronal viability that might follow after reduction of FoxP2, we used the neuronal marker Hu to determine neuronal densities in Area X 30 d after injecting either shFoxP2-f/-h or shControl virus (Figure S4). Neuronal densities in the infected region in Area X did not differ in knockdown and shControl-injected birds (two-tailed Mann-Whitney *U* test, *p* > 0.39; shControl, *n* = 4 hemispheres; shFoxP2-f/-h, *n* = 3 hemispheres). Density of neurons were also comparable inside and outside of the virus-infected region of Area X for all viruses (two-tailed Mann-Whitney *U* test, *p* > 0.6 for both shFoxP2-f/-h and shControl). In sum, these data demonstrate that virus-mediated RNAi can induce specific, long-lasting knockdown of gene expression in zebra finch Area X without causing cell death.

### Song Imitation of *FoxP2* Knockdown Zebra Finches

Adult zebra finch song consists of different sound elements, here called syllables, that are separated by silent intervals. Syllables are rendered in a stereotyped sequential order, constituting a motif. During a song bout, a variable number of motifs are sung in short succession. To obtain a first descriptive account of the song of knockdown and control pupils, we measured mean acoustic features for all syllables recorded from all pupils using the software Sound Analysis Pro (SAP) [22]. The features extracted were mean pitch, mean frequency, mean frequency modulation (FM; change of frequency in time), mean entropy, and mean pitch goodness (PG; periodicity of sound), as well as mean duration. The comparison of the distribution of these features across the repertoire of knockdown and control pupils did not reveal any significant differences, indicating that knockdown pupils, control pupils, and tutors sang syllables with similar acoustic features (Figure S5).

**Figure 2:**
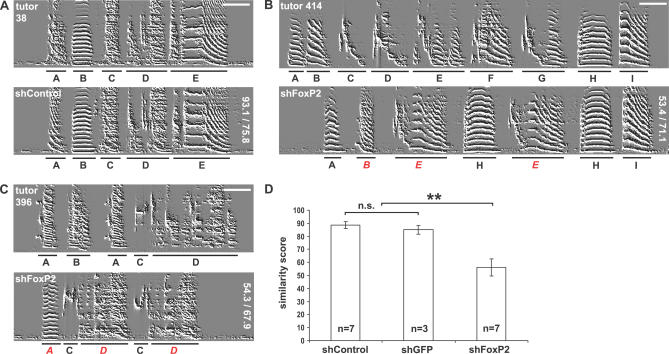
Incomplete Tutor Song Imitation by *FoxP2* Knockdown Pupils Each sonogram depicts a typical motif of one animal (scale bars indicate 100 ms, frequency range 0–8,600 Hz). Tutor syllables are underlined with black bars and identified by letters. The identity of pupil syllables was determined by similarity comparison to tutor syllables using SAP software. Imprecisely copied pupil syllables are designated with red italic letters. (A–C) (A) tutor #38 and shControl-injected pupil, (B) tutor #396 and shFoxP2-injected pupil, and (C) tutor #414 and shFoxP2-injected pupil. ShFoxP2-injected pupils copied fewer syllables and the fidelity of syllable imitation was worse than in shControl pupils, reflected by lower SAP scores (similarity/accuracy indicated vertically at the right edge of the sonograms). (D) The mean similarity scores between tutor and pupil motifs were significantly lower in shFoxP2- injected pupils than in shControl- and shGFP-injected pupils (± SEM; two-tailed Mann-Whitney *U* test, **p < 0.001, Bonferroni-corrected α-level). There was no significant difference between shGFP- and shControl-injected animals (not significant [n.s.], *p* > 0.5).

**Figure 3:**
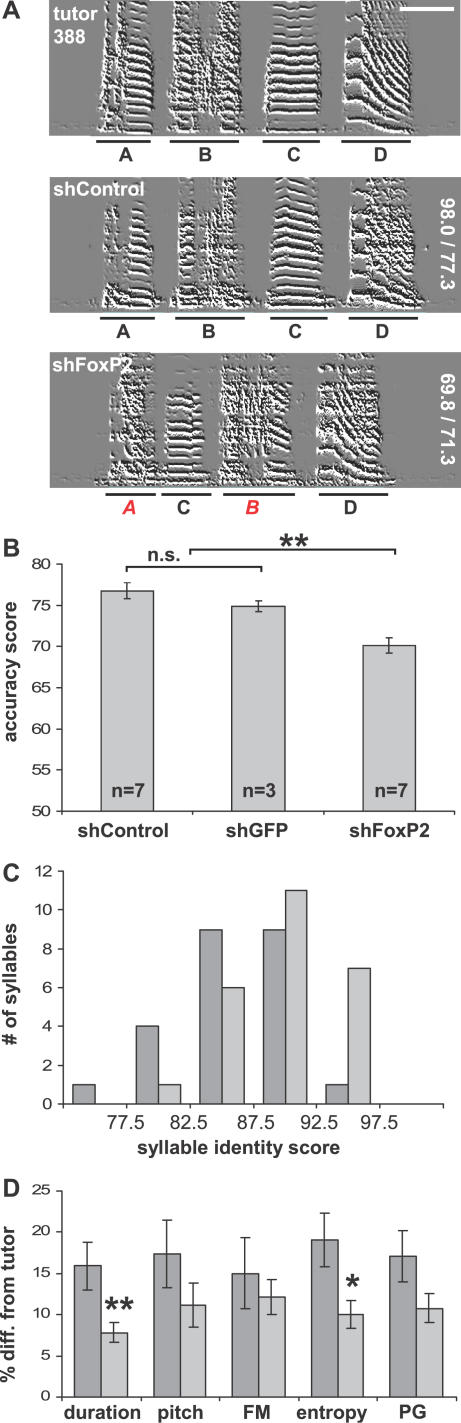
Inaccurate Tutor Song Imitation by *FoxP2* Knockdown Pupils (A) Representative sonograms of *FoxP2* knockdown and control pupils both tutored by male 388 (scale bars indicate 100 ms, frequency range = 0–8,600 Hz). Syllables are underlined with black bars and identified by letters. The identity of pupil syllables was determined by similarity comparison to tutor syllables using SAP software. Red italic letters denote imprecisely copied syllables. Inaccurate imitation is particularly evident in the second element of syllable A and the first element of syllable B. Similarity and accuracy scores are indicated vertically at the right edge of the sonograms. (B) Average motif accuracy was significantly lower in shFoxP2 knockdown pupils compared to control pupils, indicating that they imitated their tutors less precisely (±SEM; two-tailed Mann-Whitney *U* test, **p < 0.001, Bonferroni-corrected α-level). shControl- and shGFP-injected pupils copied their tutors with similar precision (n.s., *p* > 0.4). (C) The frequency distribution of identity scores of all syllables from *FoxP2* knockdown pupils (dark grey bars)was shifted towards lower scores, compared to control pupils (light grey bars).. This suggests that all syllable types were affected. (Identity scores were obtained from comparison of pupil/tutor syllable pairs; shFoxP2 *n* = 24 syllables from 7 animals; shControl *n* = 26 syllables from 7 animals). (D) Comparison of syllable duration and mean acoustic feature values (FM and PG) between pupil syllables and their respective tutor syllables. The divergence of imitated syllables from the tutor model tended to be larger for all acoustic measures in the *FoxP2* knockdown pupils (dark grey bars) than in the controls (light grey bars). For average syllable duration and mean entropy measures, the difference was significant (±SEM; two-tailed Mann-Whitney *U* test, **p < 0.001 for duration and *p < 0.05 for entropy; Bonferroni-corrected α-level; shFoxP2 *n* = 7 animals, on average 3 syllables per animal; shControl *n* = 7 animals, on average 4 syllables per animal).

Next, we analyzed the behavioral consequences of bilateral *FoxP2* knockdown in Area X for the outcome of song learning at PHD90. When a juvenile male finch is tutored individually by one adult male, the pupil learns to produce a song that strongly resembles that of his tutor [23]. We therefore determined learning success by the degree of acoustic similarity between pupil and tutor songs. Analysis of song recorded at PHD90 revealed that pupils with experimentally reduced FoxP2 levels in Area X imitated tutor songs with less fidelity than control animals did (see also Audio S1–S6). The comparison of sonograms from shControl-injected (Figure 2A) and shFoxP2-injected pupils (Figure 2B and 2C) with their respective tutors shows the characteristic effects caused by reduction of FoxP2. Typical features of *FoxP2* knockdown pupils included syllable omissions (Figure 2B, syllables C, D, F, and G; Figure 2C, syllable B), imprecise copying of syllable duration (Figure 2B, syllable E longer; Figure 2C, syllable D shortened), and inaccurate imitation of spectral characteristics (Figure 2B, syllable E; Figure 2C, syllable D). In addition, in four out of seven knockdown pupils, the motif contained repetitions of individual syllables or syllable pairs (e.g., see Figure 2B and 2C). In contrast, none of the control or tutor motifs contained repeated syllables. Pupils did not reverse the sequential order of syllables in the tutor motifs, except for one control (unpublished data) and one *FoxP2* knockdown pupil (Figure 3A).

Acoustic similarity between pupil and tutor song was measured with SAP by pairwise comparison of user-defined pupil and tutor motifs. SAP provides a similarity score that indicates how much of the tutor sound material was imitated by the pupil, regardless of syllable order. The distinction between imitated and non-imitated sounds in SAP is based on *p*-value estimates derived from the comparison of 250,000 sound interval pairs, obtained from 25 random pairs of zebra finch songs (see Materials and Methods and [22] for further details). The similarity score was significantly lower in *FoxP2* knockdown than in control animals (Figure 2D). In addition, we also manually counted the number of user-defined syllables copied from the tutors, confirming that knockdown animals imitated fewer syllables (Figure S6).

Even though knockdown animals copied tutor syllables, their imitation appeared to be less precise than in control animals. Figure 3A illustrates the inaccurate syllable imitation (syllables A and B) in a knockdown pupil that learned from the same tutor as the shControl-injected pupil shown. To quantify how well the syllables of a motif were imitated on average, we obtained motif accuracy scores in SAP from pairwise motif comparisons between pupil and tutor. The motif accuracy score measures the extent to which the pupil's sounds are closer to the tutor than expected by chance. The average accuracy per motif was significantly lower in knockdown pupils than in shControl-injected pupils (Figure 3B). Of note, both shFoxP2 hairpins (shFoxP2-f and shFoxP2-h) affected motif similarity and motif accuracy scores to a similar degree (Figure S7), which is consistent with their comparable efficiency in reducing *FoxP2* mRNA in vitro (Figure 1F). Neither the similarity score nor the accuracy score correlated with the volume of Area X targeted in the pupil. Possibly, there were too few values to observe such a correlation or the absolute volume targeted by shFoxP2 virus has only a small influence on the outcome of learning.

To investigate whether inaccurate imitation affected all or only some syllables, we compared corresponding syllable pairs between tutors and pupils using a syllable identity score. The syllable identity score reflects both the degree of similarity (i.e., quantity of imitation) and the degree of accuracy (i.e., quality of imitation) in a single measure. The frequency distribution of identity scores of all syllables from *FoxP2* knockdown pupils was shifted towards lower scores compared to control pupils. This suggests that imprecise imitation was not skewed towards particular syllables or syllable types (Figure 3C), pointing to a generalized lack of copying precision. Consistent with the reduced accuracy of motif imitation (Figure 3B), we also found that syllable identity scores were significantly lower in knockdown pupils compared to control pupils (syllable identity score averaged for each animal, two-tailed Mann-Whitney *U* test, *p* < 0.02; *n* = 7 for both shFoxP2 and shControl).

To rule out that the lower imitation success of knockdown animals was related to specific song characteristics of the tutors or their lacking aptitude for tutoring, we used some males to tutor both knockdown and control pupils. Direct comparison of the motif similarity and accuracy scores from control and knockdown pupils tutored by the same male revealed significantly lower scores for knockdown compared to control pupils (average similarity score 82.6 ± 3.6 for shControl and 61.9 ± 5.6 for shFoxP2; average accuracy score 73.8 ± 0.7 for shControl and 71.7 ± 0.4 for shFoxP2; ± SEM; *n* = 5, two-tailed Mann-Whitney *U* test, *p* < 0.03 for similarity and *p* < 0.03 for accuracy; see also Figure 3A).

Because the shControl hairpin, in contrast to shFoxP2-f/-h, has no target gene, it might not stably activate the RNA-induced silencing complex (RISC) essential for knockdown of gene expression during RNAi. Because recent work suggests an involvement of the RISC in the formation of long-term memory in the fruitfly [24] we addressed a possible influence of RISC activation during song learning. For this, we compared song imitation in shGFP virus–injected pupils, in which virally expressed GFP is lastingly knocked down (Figure 1J), and shControl-injected pupils. Similarity and accuracy scores did not differ significantly between shGFP-injected and shControl-injected animals, ruling out that RISC activation contributed to the effects of shFoxP2 on song imitation (Figures 2D and 3B).

Finally, we investigated the precision of syllable imitation on the level of individual acoustic features by comparing the mean values of acoustic features of pupil syllables to those of their respective tutor. The divergence of imitated syllables from the tutor tended to be larger in all acoustic measures in the *FoxP2* knockdown pupils than in the controls. For average syllable duration and mean entropy measures, the difference was significant (Figure 3D).

### Song Performance of *FoxP2* Knockdown Zebra Finches

Area X is part of a basal ganglia–forebrain circuit, the anterior forebrain pathway (AFP), which bears similarities with mammalian cortical–basal ganglia loops [25]. The pallial target of the AFP, nucleus lateral magnocellular nucleus of the nidopallium (lMAN), may act as a neural source for vocal variability in juvenile zebra finches [13,26]. Similarly, in adult zebra finches, neural variability in AFP outflow is associated with the variability of song [27], and experimental manipulations inducing adult song variability require an intact AFP [28,29]. To explore AFP function in *FoxP2* knockdown and control zebra finches, we investigated the variability of their song syllables.

**Figure 4:**
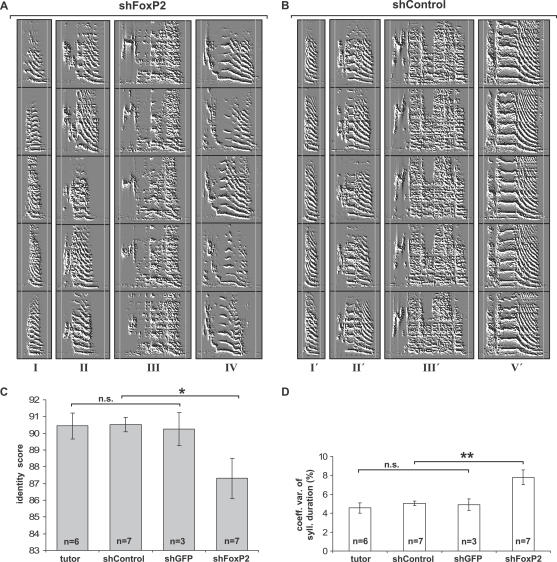
Variability of Syllable Production in *FoxP2* Knockdown Pupils Each vertical column shows the sonograms of five different renditions of the same syllable (scale bar indicates 100 ms, frequency range = 0–8,600 Hz). Each syllable, labeled by a roman numeral, was selected from a different bird. Of note, the first three syllables in (A) (syllables I, II, and III) were imitated from the same tutor as the corresponding syllables in (B) (syllables I′, II′, and III′). (A) *FoxP2* knockdown pupils. Two vertical lines mark the beginning and the end of the longest rendition of each syllable to visualize variability of syllable duration (particularly evident in syllables II and IV). Also note the variability in acoustic structure between different renditions of the same syllable (e.g., FM of syllable I, shape and frequency of first element of syllable III, and PG of last element of syllable IV). (B) Syllable duration is relatively invariant in control pupils, as indicated by the vertical lines marking the beginning and the end of each syllable. Acoustic structure is also stable across syllable renditions. (C) Acoustic variability of syllables from rendition to rendition was higher in shFoxP2-injected than in control pupils (shGFP and shControl injections), as indicated by significantly lower syllable identify scores (two-tailed Mann-Whitney *U* test, *p < 0.05; Bonferroni-corrected α-level). Control and tutor birds sang with comparable variability (two-tailed Mann-Whitney *U* test, *p* > 0.8; tutors *n* = 6, on average 5 syllables per animal; shControl *n* = 7 animals, on average 4 syllables per animal; shGFP *n* = 3, on average 5 syllables per animal). (D) Syllable duration varied more from rendition to rendition in knockdown pupils (shFoxP2) than in controls (shControl and shGFP) and tutors, as indicated by a higher mean coefficient of variation of syllable duration (±SEM, two-tailed Mann-Whitney *U* test, **p < 0.001; Bonferroni-corrected α-level; no difference between tutors, shControl-injected, and shGFP-injected animals, *p* > 0.7, same animals as [C]).

The comparison of sonograms from different renditions of the same syllable revealed that knockdown pupils sang their syllables in a more variable fashion than control pupils (Figure 4A and 4B). Both the spectral (syllables I and III) and the temporal domain (syllables II and IV) were affected. Of note, the first three syllable examples shown in Figure 4A and 4B (syllables I, II, and III and I′, II′, and III′), stem from different animals, but were learned from the same tutor. To quantify the acoustic variability of syllables, we used the syllable identity score mentioned above. Pairwise comparison between different renditions of the same syllable revealed that shFoxP2-injected pupils sang syllables slightly, but significantly, more variably than control pupils or tutors (Figure 4C). As expected, shControl-injected pupils, shGFP-injected pupils, and tutors performed their syllables with equal stability (Figure 4C).

Next, we quantified the variability of syllable duration between different renditions of the same syllable. The coefficient of variation of syllable duration was significantly higher in knockdown than in control pupils and tutors, suggesting imprecise motor coordination on short temporal scales (Figure 4D). Notably, the timing of syllables in control pupils (shControl and shGFP) was as stable as in tutors (Figure 4D). The variability of syllable duration in tutor and control birds varied in the same range as reported previously [30], emphasizing how tightly adult zebra finches normally control syllable duration.

Finally, we analyzed the sequential order of syllables over the course of many motifs. To this end, we first annotated sequences of 300 user-defined syllables with the positions in their respective motifs. We then measured the stereotypy of a motif by calculating for each syllable the entropy of its transition distribution. Based on this entropy measure, we generated a sequence consistency score (1 − entropy), which reflects song stereotypy. An entropy score of 0 indicates random syllable order, whereas a score of 1 reflects a fixed syllable order. The mean sequence consistency was similar in shControl and shFoxP2-f/-h animals (Figure S8). Because stereotypy of motif delivery is a hallmark of “crystallized” adult song, it seems plausible that both knockdown animals and controls had reached the end of the sensory-motor learning period [31]. To investigate this question in more detail, we next analyzed the song of knockdown and control pupils recorded at earlier stages of song development.

### Song Development in *FoxP2* Knockdown Zebra Finches

**Figure 5:**
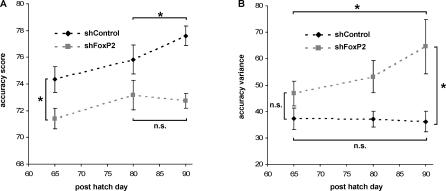
Differences in Song Development of *FoxP2* Knockdown and Control Pupils (A) We measured the accuracy of syllable imitation in song recordings of the same pupils made at three different ages (PHD65, PHD80, and PHD90), using the automated batch procedure in Sound Analysis Pro. Data points represent mean values (±SEM) of 1,000–3,000 pairwise comparisons between pupil recordings and the tutor model (shFoxP2-f/-h, *n* = 7 animals for all ages; shControl, *n* = 5 animals for PHD65, *n* = 6 animals for PHD80, and *n* = 7 animals for PHD90). Syllable imitation was already less accurate in *FoxP2* knockdown pupils by 65PHD (two-tailed Mann-Whitney *U* test, PHD65 *p < 0.05), and does not continue to improve beyond 80 d, in contrast to control pupils (two-tailed Wilcoxon signed-rank test, PHD80 to PHD90 *p < 0.05 in shControl, n.s. in shFoxP2-f/-h, *p* > 0.5). The dashed line connecting the data points illustrates the directionality of changes over time. but does not imply a linear relationship. (B) Variance of syllable accuracy values increased with age in knockdown pupils, but not in controls (two-tailed Wilcoxon signed-rank test, PHD65 to PHD90 *p < 0.05 in shFoxP2-f/-h, n.s. in shControl, *p > 0.4). This leads to significantly higher variance at PH90 in knockdown pupils compared to control pupils (two-tailed Mann-Whitney *U* test, PHD90 *p < 0.05). Dashed lines as in (A).

To explore the developmental trajectory of song learning in knockdown and control pupils, we analyzed songs recorded during plastic song at PHD65 and towards the end of the learning phase at PHD80. Since syllables are not yet rendered in a stereotyped motif structure at PHD65, we quantified song imitation success and vocal variability on the level of the syllables only. To avoid the necessity of identifying individual syllables based on their morphology, we made use of an automated procedure provided by SAP to compare all song material from a given day to the tutor's typical motif. The vocalizations of pupils were first segmented into syllables. All segments were subsequently compared to the typical motif of the tutor in a pairwise fashion (between 1,000–3,000 comparisons per pupil per day). The output variable of these measurements is an accuracy score, which describes the extent to which the pupil's sounds match those of the tutor (see Materials and Methods and [22] for further details). We found that knockdown pupils imitated their tutors less accurately than control pupils already at PHD65 (Figure 5A). The frequency distribution of accuracy values also suggests that imprecise syllable imitation was not skewed towards particular syllables or syllable types (Figure S9). This result is in line with the observation made earlier for the syllables at PHD90 (Figure 3C). In contrast to control pupils, knockdown pupils did not improve in accuracy after PHD80, suggesting they had reached the end of the learning phase (Figure 5A).

For each pupil, we also calculated the change of accuracy from one age to the next (accuracy [age*_n_* − age*_n_*_−1_]). The change of accuracy from PHD65 to PHD80 was indistinguishable between knockdown and control pupils (two-tailed Mann-Whitney *U* test, *p* > 0.9; *n* = 5 for shFoxP2-f/-h and *n* = 7 for shControl), suggesting that up to this age, syllable imitation followed largely similar dynamics. However, from PHD80 to PHD90, accuracy of syllable imitation continued to improve only in control, but not in knockdown pupils (two-tailed Mann-Whitney *U* test, *p* < 0.04; *n* = 6 for shFoxP2-f/-h and *n* = 6 for shControl).

In order to investigate variability of syllable production during song development, we compared the variance of accuracy values between knockdown and control pupils. Whereas the variance was similar between the two experimental groups at PHD65 and at PHD80, it was significantly higher in knockdown pupils compared to controls at PHD90 (Figure 5B). This difference resulted from an increase of variance with age in shFoxP2-injected birds (Figure 5B). Of note, the similarity batch analysis, which does not require assumptions about the identity of individual motifs or syllables, confirmed the results on both lower imitation success and higher vocal variability obtained in our prior analysis of the songs from PHD90 (Figures 2D and 3B).

## Discussion

Our goal was to investigate the requirement of *FoxP2* for normal song development in the zebra finch, a model for studying the basic principles of vocal learning. To this end, we analyzed the behavioral consequence of an experimental reduction of *FoxP2* during song development. Using lentivirus-mediated RNAi for the first time in the songbird brain, we reduced *FoxP2* mRNA and protein levels in Area X with either of two different knockdown constructs. We found that this prevented complete and accurate imitation of the tutors' song, an effect already evident during plastic song. Reduced *FoxP2* levels also led to more variable performance of syllables in adults. In contrast, we observed no such abnormalities in birds with Area X injections of virus knocking down an exogenously expressed gene (*GFP*) or expressing a nontargeting control construct. In addition, we verified in vitro that knockdown of *FoxP2* did not affect protein levels of FoxP1, the closest homolog of *FoxP2. FoxP2* knockdown also did not cause apoptotic cell death in Area X, and it did not alter the density of neurons in this nucleus. Consistent with this, *FoxP2* knockdown pupils showed different song abnormalities than birds with electrolytic lesions in Area X. Juvenile Area X lesions result in low sequence consistency, and the repertoire of birds with juvenile Area X lesions contains unusually long syllables [13], which were not observed in *FoxP2* knockdown finches (Figure S5). Together, these data rule out that unspecific effects of RNAi induction, viral infection, or damage to Area X influenced our results. We further eliminated the possibility that specific song features of the tutor birds contributed to the behavioral differences. The outcome of song learning was affected by virus infection in approximately 20% of the volume of Area X. This result is consistent with a previous study on virally injected rats, in which blocking neural plasticity in 10%–20% of lateral amygdala neurons was sufficient to impair memory formation [32]. Taken together, these data strongly suggest that insufficient levels of FoxP2 in Area X spiny neurons lead to incomplete and inaccurate vocal imitation, implicating *FoxP2* in postnatal brain function.

The incomplete and inaccurate vocal imitation of tutor song in *FoxP2* knockdown pupils raises the question whether knockdown pupils were unable to generate particular sounds. Given that syllables with similar spectral features could be learned or omitted by the same pupil (e.g., in Figure 2B, tutor syllables E and G are similar; pupil imitated E, but not G), this does not seem likely. Also, omitted syllables did not differ in their spectral feature composition from those that were learned by knockdown animals (unpublished data). Consistent with this, the distributions of mean syllable feature values and mean duration across the syllable repertoire were indistinguishable between knockdown and control pupils (Figure S5). However, it is still possible that *FoxP2* knockdown affected the motor control of singing. The fact that *FoxP2* knockdown pupils produced syllables more variably than controls at PHD90 would be consistent with this. Importantly though, this increased variability of syllable rendition in *FoxP2* knockdown pupils was not yet evident at PHD65, when tutor imitation was already less proficient (Figure 5B). Thus, the increased syllable variability is apparently not causally related to the observed tutor imitation deficit. Unfortunately, song analysis alone cannot ultimately distinguish between impairments in motor production and motor learning. Any motor production deficit likely affects the auditory feedback signal, which in turn is bound to reduce the quality of tutor imitation. Knockdown of FoxP2 in adult zebra finches might help to clarify the contribution of FoxP2 to motor control.

Although knockdown animals were apparently not unable to produce particular syllable types, given the involvement of the basal ganglia in the acquisition and performance of motor sequences [33], knockdown pupils might have been impaired in producing particular sequences of syllables, i.e., in moving from one syllable to the next. We found that knockdown pupils could in principle imitate adjacent tutor syllables in the same order (e.g., Figure 2B, syllables A and B, and H and I; Figure 2C, syllables C and D). There was also no preferred position (i.e., beginning or end of song) for imitated and non-imitated syllables. Moreover, potential sequencing problems might occur at different syllable transitions within the motif or intermittently in different renditions of the motif. Both scenarios would result in low sequence stereotypy, which we did not find (Figure S8). The limited imitation success of *FoxP2* knockdown pupils could also result from an imprecise neural representation of the tutor model. There is evidence for an involvement of Area X in sensory learning at PHD35 [34], but the up-regulation of FoxP2 in Area X at PHD50 and PHD75 rather speaks for an involvement of FoxP2 in sensory-motor learning [10].

Under the assumption of a model of reinforcement-based motor learning mediated through the basal ganglia, the animal initially generates variable motor output. Progressively, particular motor actions are reinforced [33]. In view of this model, *FoxP2* knockdown pupils might have either experienced a limitation in generating enough sound variability or difficulties with reinforcing the “right” motor patterns, a possibility that includes both difficulties in detecting similarity to the target or adjusting song appropriately. Since knockdown pupils sing as variable as control pupils early during song development and even more variable as adults (Figures 4 and 5B), we favor the hypothesis that knockdown pupils were impaired in adjusting their motor output according to the memorized tutor model in the course of song learning. This hypothesis is supported by the phenotypic overlap of song deficits observed in *FoxP2* knockdown pupils and birds that were prevented from matching vocal output with memorized tutor song. For instance, perturbed auditory feedback provokes syllable repetitions [35], and deafening in juveniles brings about syllables with large acoustic variability [36]. Although we cannot ultimately rule out the possibility that the impairment observed after *FoxP2* knockdown in juvenile birds was primary motor in nature, an interpretation involving a deficit with auditory-guided motor learning seems more consistent with the knockdown song phenotype.

What is the mechanism by which FoxP2 contributes to song development? In Area X, spiny neurons receive pallial glutamatergic input from Area X–projecting neurons in HVC [37]. These neurons process auditory information and are active during singing [38,39]. FoxP2 expressing spiny neurons also receive nigral dopaminergic input [10,40]. As has been suggested for motor learning in mammals [41], midbrain dopaminergic activity could act as reinforcement signal during song learning. Therefore, the integration of pallial and dopaminergic signals provides a candidate mechanism for tuning the motor output to the tutor model during learning. The increase of FoxP2 expression in Area X of zebra finches during times of vocal plasticity could be functionally related to this process. FoxP2 might mediate adaptive structural and functional changes of the spiny neurons while the song is learned. During the seasonal phase of vocal plasticity in canaries, increased *FoxP2* expression in the fall months might similarly be involved in seasonal song modifications. Since FoxP2 is a transcription factor, it could act by positively or negatively regulating plasticity-related genes. If FoxP2 functions as a plasticity-promoting factor, knockdown pupils should have been less plastic during learning, resulting in impoverished imitation and abnormally invariant song. Syllable omissions of *FoxP2* knockdowns are consistent with this notion, but more variable syllable production is clearly not. Alternatively, if FoxP2 restricts neuronal plasticity, knockdown pupils should sing more variable song. In fact, this is the case, but syllable omissions are not easily explained then. The identification of the downstream target genes of FoxP2 and the electrophysiological characterization of spiny neurons with reduced FoxP2 levels will shed light on the mechanisms by which FoxP2 affects the outcome of vocal learning.

The vocal behavior of *FoxP2* knockdown zebra finches offers a new interpretation of the speech abnormality in individuals with genetic aberrations of *FOXP2* [5], possibly extending to apraxia of speech in general [42]. The human core deficit affects the production of rapid, sequential mouth movements, which are required for speech articulation [43], and is thought to be caused by erroneous brain development. Perhaps the speech impairment results from a problem with motor learning rather than motor performance during speech learning, a hypothesis that is in line with recent theories on basal ganglia dysfunction in various developmental disorders [44]. Our results extend the similarities between learned birdsong and human speech to the molecular level, emphasizing the suitability of songbirds for investigating the basic principals of speech and its pathologies. It will be interesting to test, whether “dyspraxic song” is also perceived as different by other finches and interferes with communication, as DVD does in humans. Given female songbirds' preference for well-learned, experimentally unaltered song [45,46], we would expect this to be the case. Finally, the fact that a reduction of FoxP2 affects the outcome of both song learning and speech development provides further evidence for the hypothesis [4,21] that during evolution, ancestral genes and neural systems were adapted in the human brain and gave rise to the uniquely human capacity of language.

## Materials and Methods

### Nomenclature.

For *FoxP2* nomenclature, we followed the convention proposed by the Nomenclature Committee for the forkhead family of genes (*FOXP2* in Homo sapiens, *Foxp2* in Mus musculus, and *FoxP2* in all other species, including zebra finches) [47]. Proteins are in roman type, genes and RNA in italics.

### Generation of lentivirus.

Initially, we designed eight different constructs for the expression of short hairpin RNA (shRNA) targeting the zebra finch *FoxP2* mRNA. All *FoxP2* target sequences were located within the minimum common sequence of all isoforms (ORF of isoform IV), thus targeting all *FoxP2* isoforms described in [10]. In order to minimize potential cross-reactivity of the hairpins, we chose target sequences that contained at least six dissimilar bases with *FoxP1*, the closest homolog of *FoxP2*. and were not located within the highly conserved forkhead box domain of *FoxP2*. This shRNA design is stringent in comparison to a recently published guideline [48] that recommends including at least three mismatches to untargeted sequences. The structure of the linear DNA encoding shRNA hairpins was sense-loop-antisense. The sequence of the loop was GTGAAGCCACAGATG. Each hairpin construct was tested for knockdown efficiency in HEK293 T cells in vitro by simultaneous overexpression with zebra finch *FoxP2*, tagged with the V5 epitope. Subsequent western blot analysis using a V5 antibody revealed two hairpins (shFoxP2-f, target sequence AACAGGAAGCCCAACGTTAGT, and shFoxP2-h, target sequence AACGCGAACGTCTTCAAGCAA) that strongly reduced *FoxP2* expression levels. To demonstrate the sequence specificity of the hairpins to the *FoxP2* gene, we also simultaneously overexpressed them with *FoxP1*, cloned from adult zebra finch brain cDNA and tagged with the V5 epitope. The DNA fragments encoding the hairpins shFoxP2-f and shFoxP2-h were subcloned into a modified version of the lentiviral expression vector pFUGW [17] containing the U6 promoter to drive their expression. To use as controls, we subcloned fragments encoding a hairpin targeting GFP (shGFP, target sequence GCAAGCTGACCCTGAAGTTCA) and a nontargeting hairpin (shControl, sequence AATTCTCCGAACGTGTCACGT) into the modified pFUGW. All viral constructs expressed GFP under control of the human ubiquitin C promoter. Recombinant lentivirus was generated as described in [17]. Titers were adjusted to 1–2 × 10^6^/μl.

### Stereotaxic injection of virus.

The general procedure for studying the behavioral consequences of locally reduced *FoxP2* levels in Area X was as follows (Figure S1). Young zebra finches from our colony at the Max-Planck-Institute for Molecular Genetics were sexed as described [49] at approximately PHD10. By PHD20, fathers and older male siblings were removed from family cages to prevent experimental zebra finches from instructive auditory experience prior to the onset of tutoring. At PHD23, animals were anaesthetized with xylazine/ketamine and stereotaxically injected with recombinant lentivirus. The stereotaxic coordinates for Area X injections were anterior/posterior 3.6 and 4.0, medial/lateral 1.4 and 1.6, and dorsal/ventral 3.8 and 4.0. Per injection site, approximately 200 nl of lentiviral solution were injected over a period of 2 min with a hydraulic micromanipulator (Narishige). On PHD30, each pupil received an adult male song tutor, and both birds were kept together for 2 mo in a sound-isolated box with automated song-recording equipment. By PHD93–95, trained pupils were perfused with 4% paraformaldehyde in 0.1 M PB and their brains dissected for histological analysis (see Figure S1 for timeline of experiments).

We determined that the virus infected FoxP2 immunopositive neurons using immunostaining as described [10]. Moreover, we used immunohistological staining with antibody Hu (1:200; Chemicon) to stain neurons and quantify the percentage of them infected by virus. Immunofluorescent sections were analyzed with a 40× oil objective, using a Zeiss confocal microscope (LSM510) with the LSM-510 software package. On average, we counted 417 virus-infected cells in five to six sections per hemisphere (seven hemispheres from five animals) and determined how many of those were also Hu^+^. We quantified the neuronal density by counting the number of Hu^+^ cells in scanning windows of 230.3 μm × 230.3 μm (two scanning windows per section) inside and outside the injection site in Area X (presented as a number of cells/mm^2^).

To identify apoptotic cells, we used a fluorescein TUNEL assay (Roche) in 50-μm sagittal sections from PHD29 male zebra finch brains, injected with shFoxP2 virus on PHD23. To increase signal intensity, we stained the sections by fluorescent immunohistochemistry with an anti-FITC antibody, followed by incubation with an Alexa568-conjugated secondary antibody. TUNEL-positive cells were counted using a fluorescence microscope. In general, the total number of TUNEL-positive cells was very low (approximately eight cells per 50-μm brain section). There was no difference between knockdown and control animals in the total number of TUNEL-positive cells.

In order to quantify the volume of Area X targeted by virus injection, we measured the area of Area X in all brain sections (thickness, 50 μm) containing it, and quantified the region visibly expressing GFP within Area X under 5× magnification on a fluorescence microscope. We then summed the values from all sections for both areas separately and calculated the ratio of GFP-positive area to total Area X, which is equivalent to the ratio of GFP-positive volume to total Area X volume. The values from left and right hemispheres were averaged per animal. In one knockdown animal, GFP expression in Area X was detected only in the right hemisphere. Since this pupil had a motif imitation score of 50.8%, which is below the range of controls (68.1 ± 2.7% mean ± SEM), but better than knockdown pupils (39.6 ± 5.0 mean ± SEM), it could be that knockdown of *FoxP2* in Area X of only the right hemisphere suffices to impair song learning consistent with right hemispheric dominance in zebra finches [50].

In six animals injected with either shFoxP2-f/-h or shControl virus, no GFP was detectable after histological analysis. We quantified imitation success in three of the six animals without GFP, and found it to be similar to zebra finches with shControl injection (similarity score = 90.7; accuracy score = 77.7; two-tailed Mann-Whitney *U* test, *p* > 0.8 for both similarity and accuracy).

### Quantification of *FoxP2* knockdown.

Young male zebra finches received an injection of shFoxP2-f/-h virus in one hemisphere and an injection with control virus (shControl) in the contralateral hemisphere on PHD23 as described above. For the quantification of protein levels after *FoxP2* knockdown, we performed an immunohistological staining with the FoxP2 antibody on 50-μm sections 30 d after virus injection. Immunohistological staining was performed as described [10], but using an antibody dilution of 1:5,000. All sections were processed at the same time with the same batch of antibody solution. Images of stained brain sections were taken with a digital camera using the Simple PCI software (Compix) at 40× magnification. For each section, we acquired multiple *Z*-stacked images of the virus-infected area (230.3 μm × 230.3 μm), and reconstructed a maximal projection. All images from the same bird were taken with the same microscope and software settings. Finally, we quantified fluorescence intensity levels in the images. The intensity of the green fluorescence from the viral GFP was not significantly different between shFoxP2-f/-h–injected and shControl-injected hemispheres (two-tailed Mann-Whitney *U* test, *p* > 0.3).

For the quantification of *FoxP2* knockdown mRNA levels, young male zebra finches were injected with shFoxP2-f/-h virus in one hemisphere and control virus (shControl) in the contralateral hemisphere on PHD23, as described above. This permitted analysis of *FoxP2* knockdown in the same bird while avoiding confounding differences in gene expression levels between birds. On PHD50, we sacrificed the birds and excised the GFP-expressing brain area with a 1-mm–diameter glass capillary (Brand) under a fluorescence dissecting microscope. RNA was extracted with TRIZOL (Invitrogen); yield was determined by UV spectroscopy at 260/280 nm with a Nanodrop device. FoxP2 expression was quantified by real-time PCR using SybrGreen (Applied Biosystems). We determined relative *FoxP2* expression levels through normalization to the expression levels of two internal control genes, which were identified in a BLAST homology search for the mouse housekeeping genes *Hmbs* and *Pfkp* in the database from the Songbird Neurogenomics Initiative (http://titan.biotec.uiuc.edu/songbird/) and the Songbird Brain Transcriptome Database (http://songbirdtranscriptome.net/). The expression of Hmbs and Pfkp in the left and right hemisphere in both injected and untreated animals was equivalent (numbers indicate fold change between left and right hemispheres; untreated: Hmbs = 1.4 ± 0.5 and Pfkp = 1.3 ± 0.6; injected: Hmbs = 1.0 ± 0.4 and Pfkp = 1.1 ± 0.4, *n*=5 birds). Relative expression levels were determined with the comparative cycle time (Ct) method. All primers used in this study amplified the cDNA with similar efficiency (*E* = 1 ± 5%) in a validation experiment. Normalized Ct values from the same animal were calibrated to the shControl-injected hemisphere. FoxP2 expression levels are thus presented as the ratio of expression in shControl- to shFoxP2 -injected hemispheres.

### Song recording and analysis.

Vocalizations were recorded between 9 am and 4 pm on PHDs 65, 80, and between 90 to 93 in absence of the tutor. Quantitative song analysis was performed using the SAP software, version 1.04 [22,51]. We analyzed song at the level of the syllables, the motif, and syntax. We define “syllable” as a continuous sound element, surrounded by silent intervals. The “typical song motif” was defined as the succession of syllables that includes all syllable types (except introductory notes), and occurs in a repeated manner during a song bout. Syntax refers to the sequence of syllables in many successive motifs.

*Motif analysis.* We quantified how well pupils had copied the motif of their tutor using a similarity score and an accuracy score obtained in SAP from ten asymmetric pairwise comparisons of the pupil's typical motif with the tutor motif. In asymmetric comparisons, the most similar sound elements of two motifs are compared, independent of their position within a motif. The smallest unit of comparison are 9.26-ms–long sound intervals (FFT windows). Each interval is characterized by measures for five acoustic features: pitch, FM, amplitude modulation (AM), Wiener entropy, and PG. SAP calculates the Euclidean distance between all interval pairs from two songs, over the course of the motif, and determines a *p*-value for each interval pair. This *p*-value is based on *p*-value estimates derived from the cumulative distribution of Euclidean distances across 250,000 sound-interval pairs, obtained from 25 random pairs of zebra finch songs. Neighboring intervals that pass the *p*-threshold value (*p* = 0.1 in this study) form larger similarity segments (70 ms). The amount of sound from the tutor's motif that was included into the similarity segments represents the similarity score; it thus reflects how much of the tutor's song material was found in the pupil's motif.

To measure how accurately pupils copied the sound elements of the tutor motif, we used the accuracy score from SAP. The accuracy score is computed locally, across short (9 ms) FFT windows and indicates how well the sound matched to the sound in the tutor song. SAP calculates an average accuracy value of the motif by averaging all accuracy values across the similarity segments.

*Syllable analysis—manual counting of imitated syllables.* For manual counting of imitated syllable types, two individuals who were blind to treatment counted all syllables that matched a tutor syllable by visual inspection of sonograms. Their interobserver reliability was 80%.

*Syllable analysis—syllable acoustic features.* We extracted the mean pitch, mean FM, mean entropy, and mean PG, as well as mean duration from 25 renditions of each syllable. To compare the similarity of individual spectral features between pupil and tutor syllables, we subtracted each mean feature value of each tutor syllable from the mean feature value of the corresponding pupil syllable. Next, we normalized the absolute differences between the values of tutor and pupil syllables to the values of the tutor syllable to obtain the difference of a pupil syllable in a given feature from the tutor syllable in percent.

To describe the variability of syllable duration between different renditions, we calculated the coefficient of variation of duration values among 25 renditions of each syllable.

*Syllable analysis—syllable identity score*. We quantified the acoustic similarity between different syllables using symmetric comparisons to obtain syllable identity scores. In contrast to asymmetric comparison, no similarity segments are identified during symmetric comparisons. Instead, the FFT windows are compared sequentially from beginning to the end of the two sounds. Thus, similarity reflects how many sound intervals were above *p*-value, and accuracy indicates the average (1 − *p*-value). To comprehensively capture the acoustic similarity between syllables in a single measure we used the product of similarity and accuracy to obtain the syllable identity score. As for the motif analysis the *p*-threshold value was set to *p* = 0.1.

To quantify how accurately pupils learned individual syllables, we performed ten symmetric comparisons of each pupil syllable with its corresponding tutor syllable. To assess how variable the same pupil performed a particular syllable in multiple renditions of his motif, we compared 20 renditions of each syllable, two at a time. Because minute temporal shifting of FFT windows is allowed in symmetric comparisons (10 ms in this study), the more variable duration of syllables in *FoxP2* knockdown animals did not bias the identity score. The syllable identity score rather reflects spectral differences between syllables.

Syntax analysis. For each pupil, we manually annotated sequences of 300 user-defined syllables with the positions in their respective motifs. That is, each syllable of a motif was given a unique integer. Based on these data, we computed the Markov chain for each pupil, i.e., all transition probabilities between syllables. To measure the stereotypy of a motif, we calculated for each syllable the entropy of its transition distribution [52]. Because motif duration differed between birds, these entropy values were rescaled by the maximal possible entropy for each given motif duration. The entropy score for a pupil was then represented by the average of these fractions of maximal entropy over all syllables. Based on this entropy measure, we generated a sequence consistency score (1 − entropy measure), which reflects song stereotypy. An entropy score of 0 indicates random syllable order, whereas a score of 1 reflects a fixed syllable order.

*Analysis of song developmen*t. To determine tutor similarity and vocal variability during plastic song and towards the end of the learning phase, we analyzed songs recorded on PHD65, PHD80, and PHD90–93 (PHD ± 1 d; in one control pupil, recordings were only available from PHD75 instead of PHD80). First, all sound files from one day were segmented into sounds in the feature batch mode of SAP. Here, the pupils' vocalization is separated from nonvocalization background using two thresholds (Wiener entropy and amplitude). The thresholds were adjusted for each pupil individually to obtain an optimal segmentation. We validated the segmentation for each pupil by visual inspection of the segments and confirmed that segments correspond to syllables. Next, all segments from a given day (between 1,000 and 3,000 segments) were automatically compared to the tutor motif. That is, in each comparison, SAP identifies the best possible match to the tutor motif for each segment. Of all segments analyzed from PHD65, PHD80, and PHD90, 11.0% ± 0.9% were less similar to the tutor model than two random zebra finch sounds are to each other, and thus did not receive any accuracy value in SAP. These sounds were found to represent cage noise, mostly. There were no differences between the amount of sounds excluded between knockdown and control pupils for any of the ages (two-tailed Mann-Whitney *U* test, *p* > 0.9 for PHD65; *p* > 0.8 for PHD80; *p* > 0.7 for PHD90).

## Supporting Information

Audio S1 Example of Song Motif from Tutor #414 (199 KB WMA)

Audio S2 Example of Song Motif from Pupil of Tutor #414 (123 KB WMA)

Audio S3 Example of Song Motif from Tutor #38 (166 KB WMA)

Audio S4 Example of Song Motif from Pupil of Tutor #38 (223 KB WMA)

Audio S5 Example of Song Motif from Tutor #396 (245 KB WMA)

Audio S6 Example of Song Motif from Pupil of Tutor #396 (370 KB WMA)

Figure S1 Timeline of Experiments By PHD20, fathers and older male siblings were removed from family cages to prevent experimental zebra finches from instructive auditory experience prior to the onset of tutoring. At the beginning of the sensory learning period at PHD23, virus was injected bilaterally into Area X. From PHD30 on, injected birds were housed individually in sound-recording chambers together with an adult male zebra finch as tutor. We recorded the song of pupils on PHD65, PHD80, and between PHD90 and 93 using an automated recording system, in absence of the tutor.
(31 KB PDF)

Figure S2 Immunohistochemical Staining with the Neuronal Marker Hu Identified Virus-Infected Neurons Expressing GFP (A) shows neuronal marker Hu, and (B) shows virus-infected neurons expressing GFP. These neurons appear yellow in the merged image (C) (scale bar indicates 20 μm).
(288 KB PDF)

Figure S3 Infection with shFoxP2-Virus Did Not Induce Apoptosis (A) We labeled apoptotic cells in 50-μm sagittal sections from PHD29 male zebra finch brains injected with shFoxP2 or shControl virus on PHD23. DNA double-strand breaks characteristic of apoptotic cells were detected using the TUNEL method, visualized with an Alexa568 secondary antibody (red). The filled white arrow points to a TUNEL-labeled cell not infected by shFoxP2-f.
(B) The open white arrow points to a shFoxP2-infected cell expressing the viral reporter GFP, but showing no TUNEL labeling (A).
(C) DAPI staining identifies cellular nuclei. The apoptotic cell (white arrow) contains fragmented DNA typical of apoptosis.
(D) Overlay picture of (A–C).
(E) As positive control for the TUNEL method, we treated a section adjacent to that shown in (A–D) for 10 min with DNAse to artificially induce DNA double-strand breaks.
(E–H) Numerous cells were now detected, among them a virally infected cell expressing GFP (white arrow in [E–H]). Colors as in (A–D).
Scale bar in (A) indicates 10 μm.
(658 KB PDF)

Figure S4 Neuronal Densities Were Similar in Area X Injected with Either shFoxP2 or shControl Neuronal densities were measured using the neuronal marker Hu in Area X 30 d after injecting either shFoxP2-f/-h or shControl virus. Bar graphs represent the number of neurons/mm^2^. Neuronal densities in the virus-infected region in Area X were similar in knockdown and shControl-injected birds (two-tailed Mann-Whitney *U* test, *p* > 0.39; shControl, *n* = 4 hemispheres; shFoxP2-f/-h, *n* = 3 hemispheres). Moreover, there were no differences between inside and outside of the injection site for any of the viruses (two-tailed Mann-Whitney *U* test, *p* > 0.6 for both shFoxP2-f/-h and shControl).
(54 KB PDF)

Figure S5 Syllables from Knockdowns and Control Zebra Finches Were Similar in the Distribution of their Acoustic Features and Their Duration Box plots represent the distribution of mean pitch (A), mean frequency (B), mean frequency modulation (FM) (C), mean entropy (D), mean goodness of pitch (PG) (E), and mean duration (F) across all syllables from tutors and each experimental group (shControl-, shGFP-, and shFoxP2-injected zebra finches). Boxes indicate the interquartile range (IQR) of the distribution; circles and asterisks specify individual values lying beyond the inner (1.5 × IQR) and outer fences (3 × IQR), respectively (*n* = 40 syllables for tutors; *n* = 31 syllables for shControl; *n* = 15 syllables for shGFP; and *n* = 31 syllables for shFoxP2). Mean syllable acoustic features and syllable duration (each averaged per animal) were not significantly different between groups (ANOVA; *n* = 6 tutors; *n* = 7 birds for shControl; *n* = 3 birds for shGFP; and *n* = 7 animals for shFoxP2-f/-h).
(60 KB PDF)

Figure S6 Manual Counting of Syllables Copied by Knockdown and Control Animals All syllables that matched a tutor syllable by visual inspection on a sonogram were counted for shFoxP2- and shControl-injected animals. Bars represent the mean percentage of tutor syllables copied by the pupils (± STDEV, two-tailed Mann-Whitney *U* test, **p = 0.004; *n* = 7 animals for both shControl and shFoxP2-f/-h).
(54 KB PDF)

Figure S7 Both Hairpin Constructs Targeting FoxP2 Affected Song Imitation to the Same Degree Bars indicate the similarity and accuracy scores, respectively, of zebra finches injected with either shFoxP2-f or shFoxP2-h (± SEM; two-tailed Mann-Whitney *U* test, *p* > 0.6 for similarity and *p* > 0.4 for accuracy).
(54 KB PDF)

Figure S8 The Syllable Sequence within Motifs Was Highly Stereotyped across Many Different Renditions, Both in shFoxP2-Injected and shControl-Injected Birds This is reflected by high sequence consistency scores (two-tailed Mann-Whitney *U* test, no significant difference between shFoxP2-f/-h and shControl, *p* > 0.6; *n* = 7 animals for both shFoxP2-f/-h and shControl). The sequence consistency score (1 − entropy) was calculated based on the entropy of sequences of 300 successive syllables.
(54 KB PDF)

Figure S9 Frequency Distribution of Syllable Accuracy Scores Was Shifted towards Lower Values in *FoxP2* Knockdown Pupils Zebra finch vocalizations recorded at PHD65 were first segmented into sounds corresponding to syllables. The segments from each bird were subsequently compared to their respective tutor motif in a pairwise fashion yielding one accuracy score for each sound segment. To obtain a balanced dataset, we randomly extracted 800 accuracy scores from each bird. Bars represent the relative frequency of accuracy scores.
(56 KB PDF)

### Accession Numbers

The GenBank (http://www.ncbi.nlm.nih.gov/Genbank) accession numbers for the genes and gene products discussed in this paper are *FoxP1* (AY549152), *FoxP2* isoform I (AY549148), *FoxP2* isoform IV (AY549151), *Hmbs* (NM_013551), and *Pfkp* (NM_019703).

The Online Mendelian Inheritance in Man (OMIM; http://www.ncbi.nlm.nih.gov/sites/entrez?db=OMIM) accession number for *FOXP2* is 605317.

## Abbreviations

AFP: anterior forebrain pathway
DVD: developmental verbal dyspraxia
FM: frequency modulation
GFP: green fluorescent protein
PG: pitch goodness
PHD: post-hatch day
RISC: RNA-induced silencing complex
RNAi: RNA interference
SAP: Sound Analysis Pro
SEM: standard error of the mean
shRNA: short interfering hairpin RNA
TUNEL: terminal deoxyribonucleotide transferase-mediated dUTP nick end labeling
