# Supplementary material for: Incomplete and Inaccurate Vocal Imitation after Knockdown of FoxP2 in Songbird Basal Ganglia Nucleus Area X
Source: PLoS Biol. 2007 Dec 4;5(12):e321. doi: 10.1371/journal.pbio.0050321 (PMC2100148; doi:10.1371/journal.pbio.0050321)
Supplement: Supplementary file 1 [file pbio.0050321.sg001.pdf]

Fig. S1

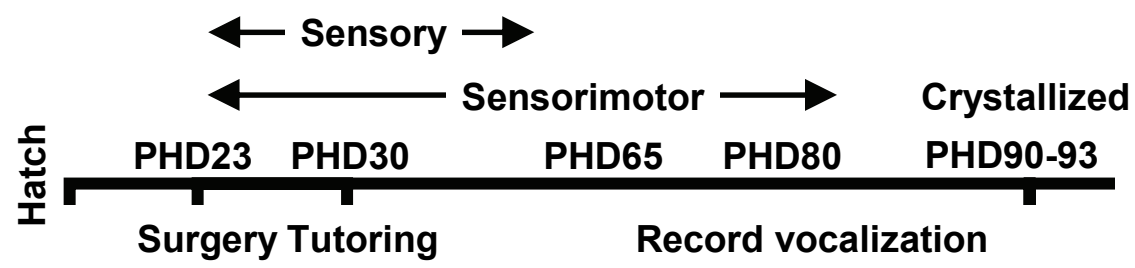

Sensory
Sensorimotor
Crystallized
Hatch
PHD23
PHD30
PHD65
PHD80
PHD90-93
Surgery
Tutoring
Record vocalization
