# Supplementary material for: Incomplete and Inaccurate Vocal Imitation after Knockdown of FoxP2 in Songbird Basal Ganglia Nucleus Area X
Source: PLoS Biol. 2007 Dec 4;5(12):e321. doi: 10.1371/journal.pbio.0050321 (PMC2100148; doi:10.1371/journal.pbio.0050321)
Supplement: Supplementary file 4 [file pbio.0050321.sg004.pdf]

Fig. S4

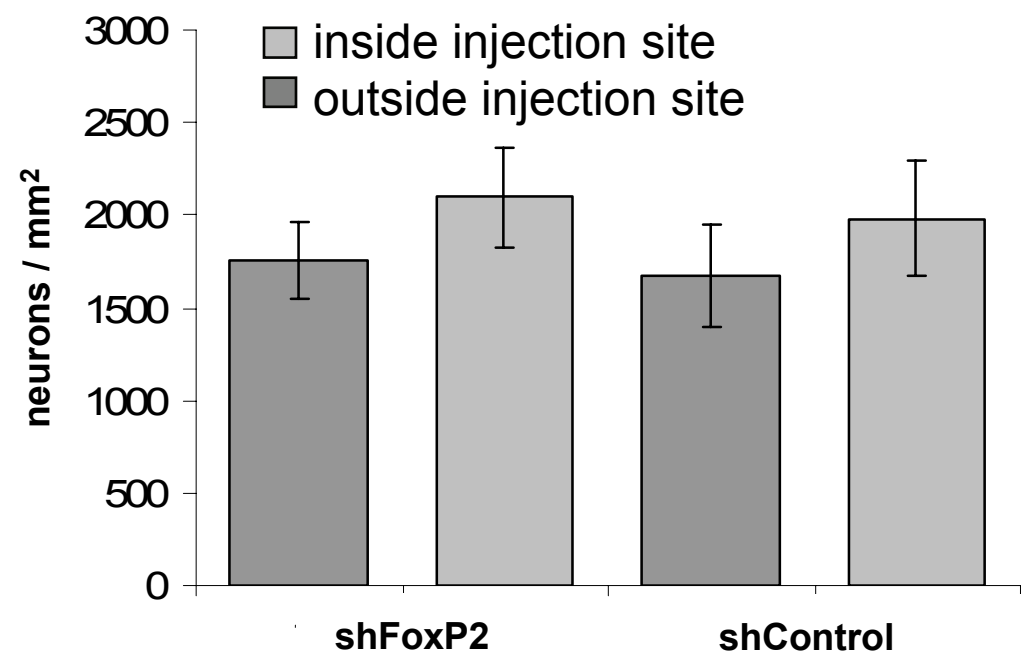

inside injection site
outside injection site
neurons / mm²
3000
2500
2000
1500
1000
500
0
shFoxP2
shControl
