# Supplementary material for: Incomplete and Inaccurate Vocal Imitation after Knockdown of FoxP2 in Songbird Basal Ganglia Nucleus Area X
Source: PLoS Biol. 2007 Dec 4;5(12):e321. doi: 10.1371/journal.pbio.0050321 (PMC2100148; doi:10.1371/journal.pbio.0050321)
Supplement: Supplementary file 5 [file pbio.0050321.sg005.pdf]

Fig. S5

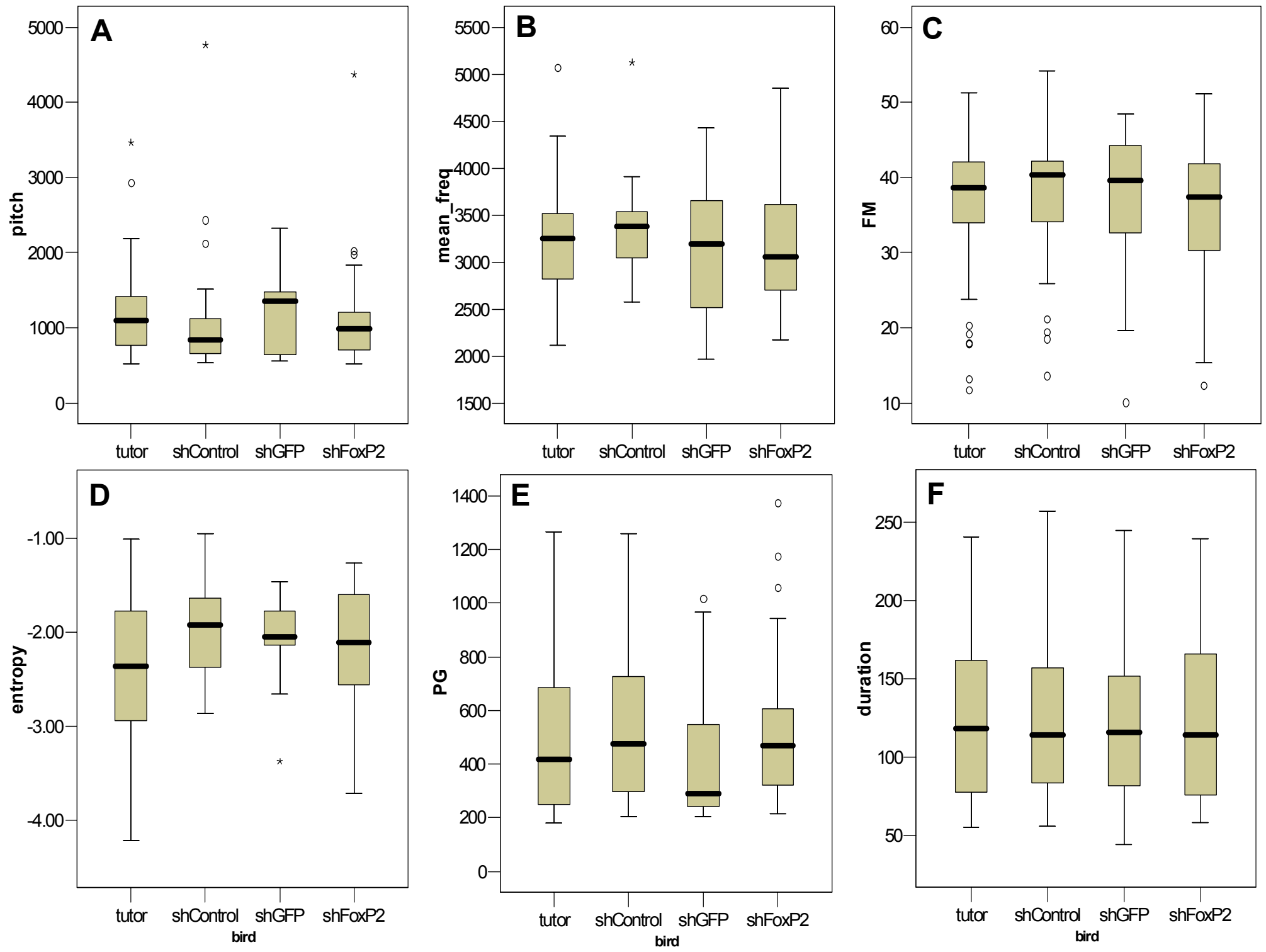

A
pitch
5000
4000
3000
2000
1000
0
tutor
shControl
shGFP
shFoxP2
B
mean_freq
5500
5000
4500
4000
3500
3000
2500
2000
1500
tutor
shControl
shGFP
shFoxP2
C
FM
60
50
40
30
20
10
tutor
shControl
shGFP
shFoxP2
D
entropy
-1.00
-2.00
-3.00
-4.00
tutor
shControl
shGFP
shFoxP2
bird
E
PG
1400
1200
1000
800
600
400
200
0
tutor
shControl
shGFP
shFoxP2
bird
F
duration
250
200
150
100
50
tutor
shControl
shGFP
shFoxP2
bird
