# Supplementary material for: Incomplete and Inaccurate Vocal Imitation after Knockdown of FoxP2 in Songbird Basal Ganglia Nucleus Area X
Source: PLoS Biol. 2007 Dec 4;5(12):e321. doi: 10.1371/journal.pbio.0050321 (PMC2100148; doi:10.1371/journal.pbio.0050321)
Supplement: Supplementary file 6 [file pbio.0050321.sg006.pdf]

Fig. S6

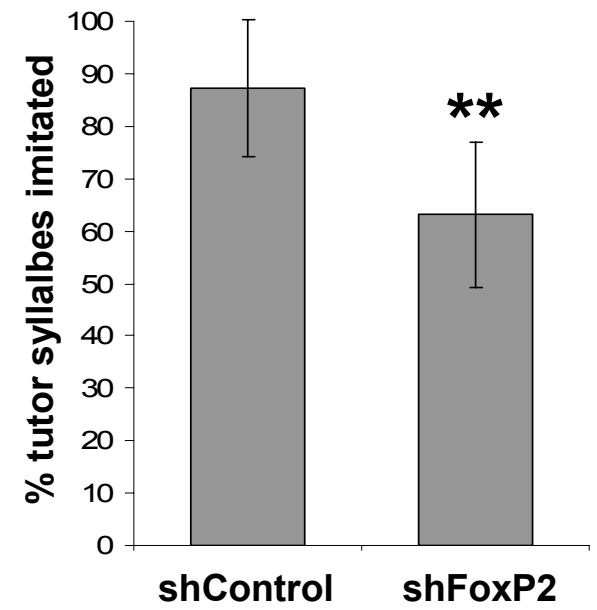

% tutor syllalbes imitated
100
90
80
70
60
50
40
30
20
10
0
**
shControl
shFoxP2
