# Supplementary material for: Incomplete and Inaccurate Vocal Imitation after Knockdown of FoxP2 in Songbird Basal Ganglia Nucleus Area X
Source: PLoS Biol. 2007 Dec 4;5(12):e321. doi: 10.1371/journal.pbio.0050321 (PMC2100148; doi:10.1371/journal.pbio.0050321)
Supplement: Supplementary file 7 [file pbio.0050321.sg007.pdf]

Fig. S7

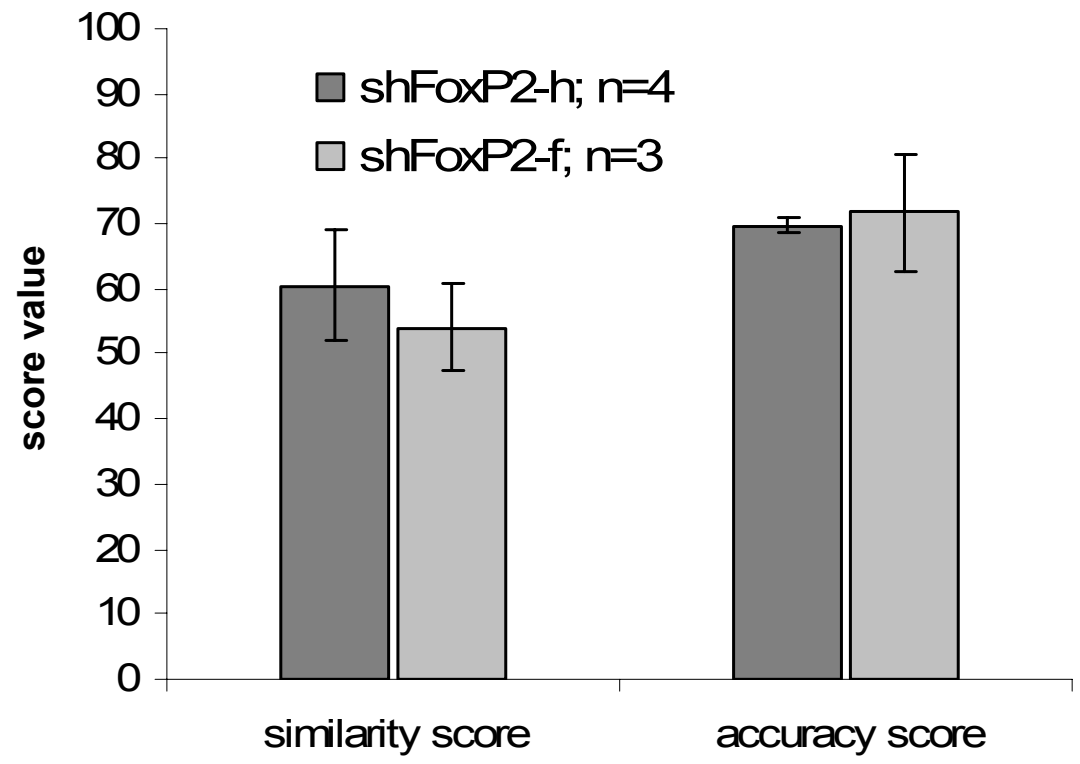
100
90
80
70
60
50
40
30
20
10
0
score value
shFoxP2-h; n=4
shFoxP2-f; n=3
similarity score
accuracy score
