# Supplementary material for: Incomplete and Inaccurate Vocal Imitation after Knockdown of FoxP2 in Songbird Basal Ganglia Nucleus Area X
Source: PLoS Biol. 2007 Dec 4;5(12):e321. doi: 10.1371/journal.pbio.0050321 (PMC2100148; doi:10.1371/journal.pbio.0050321)

Fig. S8

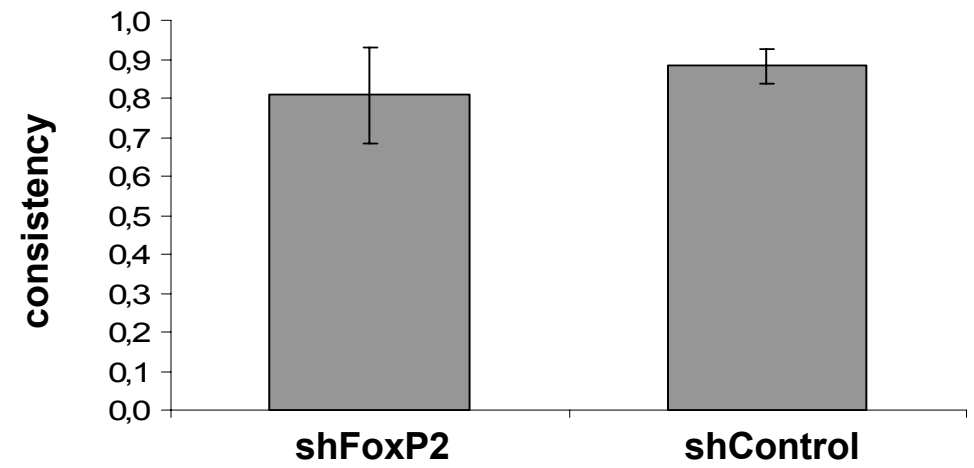

Supplement: Supplementary file 8 [file pbio.0050321.sg008.pdf]
