# Supplementary material for: Incomplete and Inaccurate Vocal Imitation after Knockdown of FoxP2 in Songbird Basal Ganglia Nucleus Area X
Source: PLoS Biol. 2007 Dec 4;5(12):e321. doi: 10.1371/journal.pbio.0050321 (PMC2100148; doi:10.1371/journal.pbio.0050321)
Supplement: Supplementary file 9 [file pbio.0050321.sg009.pdf]

Fig. S9

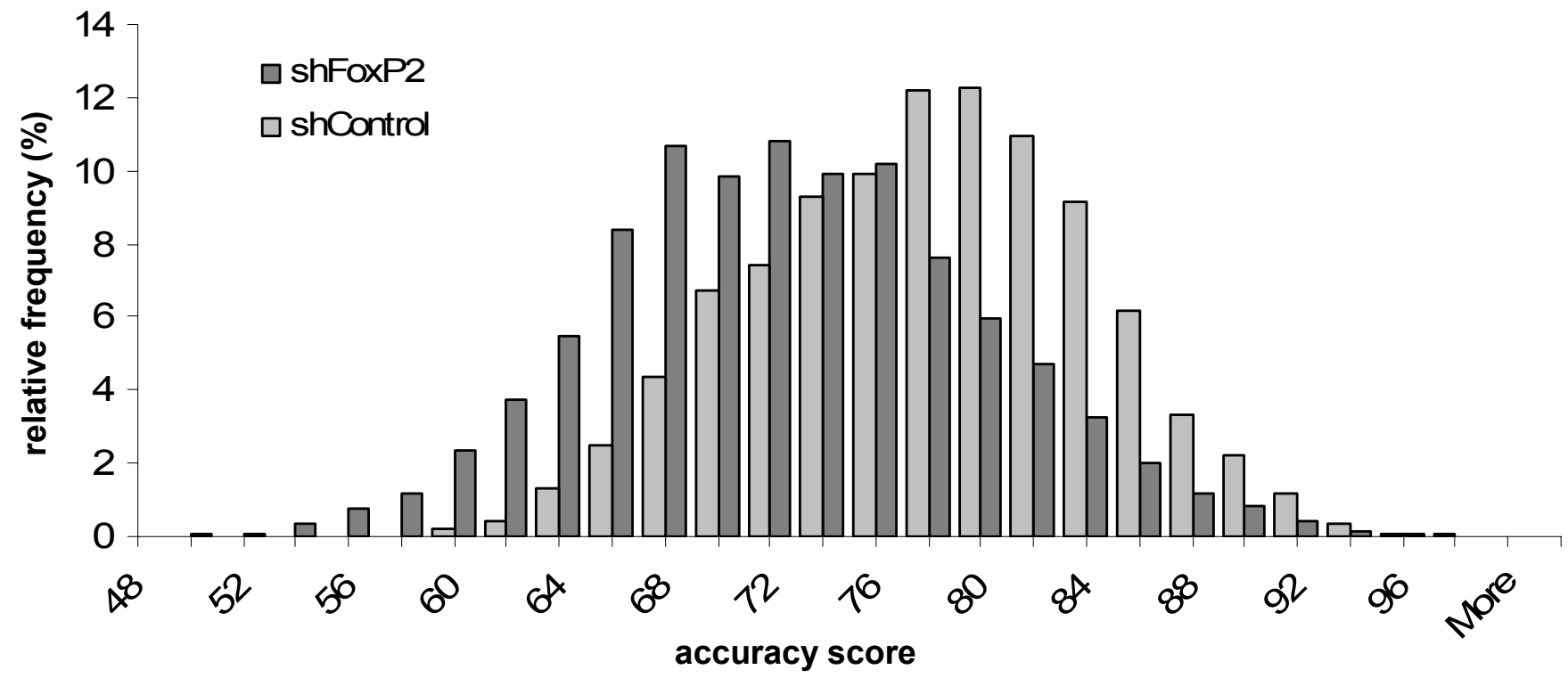
shFoxP2
shControl
relative frequency (%)
accuracy score
14
12
10
8
6
4
2
0
48
52
56
60
64
68
72
76
80
84
88
92
96
More
